# Sterile Leukocytosis Predicts Hemorrhagic Transformation in Arterial Ischemic Stroke: A National Inpatient Sample Study

**DOI:** 10.7759/cureus.14973

**Published:** 2021-05-11

**Authors:** Aldana M Antoniazzi, Santiago R Unda, Daniel M Klyde, Raphael Miller, Sharon Lam, Rose Fluss, David J Altschul

**Affiliations:** 1 Neurological Surgery, Montefiore Medical Center, New York, USA; 2 Neurological Surgery, Montefiore Medical Center, Bronx, USA; 3 Neurological Surgery, Albert Einstein College of Medicine, Bronx, USA

**Keywords:** leukocytosis, systemic inflammation, hemorrhagic transformation, stroke, nationwide inpatient sample (nis)

## Abstract

Objective: Hemorrhage transformation (HT) is a known complication of arterial ischemic stroke (AIS). In addition, it is known that the increase of proinflammatory immune cells in the brain tissue after AIS predict worse outcomes. However, it is not clear whether inflammation due to preceding or post-stroke infections affect outcomes and moreover, if systemic inflammatory markers could be useful as a clinical prediction tool for HT post-stroke. Therefore, our objective was to assess the association between systemic pro-inflammatory profile in AIS patients with HT and in-hospital mortality that did not course with acute infections during hospitalization.

Methods: This study was conducted using the 2016 and 2017 National Inpatient Sample (NIS) with International Classification of Diseases (ICD-10) codes. Multivariate logistic regression was used to examine the association between HT and in-hospital mortality with pro-inflammatory anomalies of white blood cells (WBCs) in AIS patients. Exclusion criteria comprised patients with under 18 years old, and with a diagnosis of gastrointestinal, urogenital, respiratory infection, bacteremia, viral infection, sepsis, or fever.

Results: A total of 212,356 patients with AIS were included in the analysis. 422 (0.2%) patients had a HT and 10,230 (4.8%) patients died during hospitalization. The most common WBC pro-inflammatory marker was leukocytosis with 6.9% (n=29/422) of HT and 5.5% (n=560/10,230) of patients that died during hospitalization. After adjusting for socio-demographic, comorbidities and treatment factors, leukocytosis was found to be an independent risk factor for both outcomes, HT [OR = 1.5, 95% CI: 1-2.3, p=0.024] and, in-hospital mortality [OR = 1.5, 95% CI: 1.3-1.6, p < 0.001].

Conclusion: Sterile leukocytosis is a potential clinical prediction tool to determine which patients are at higher risk of developing HT and die during hospitalization.

## Introduction

Arterial ischemic stroke (AIS) is the fifth leading cause of death in the United States [1]. Although thrombolysis is a standard treatment commonly used in acute AIS patients [2], this procedure has known complications - the major one being hemorrhagic transformation (HT), which is strongly associated with increased morbidity and mortality [3,4]. Among the pathophysiological mechanisms that promote the dynamic and complex HT phenomenon, the blood-brain barrier (BBB) disruption and brain damage driven by systemic inflammation has shown a prominent role [5].

After AIS onset, the activation of the immune cells such as leukocytes, microglia, mast cells and astrocytes release substances including cytokines, proteases and, reactive oxygen species (ROS) making the BBB more permeable and at the same time, blocking cerebral microvasculature blood flow [6-8]. Through the disrupted BBB, peripheral immune cells enter the brain parenchyma and enhance the ongoing neuroinflammatory process. Thus, in the postischemic inflammatory sequence of events involving the brain, its vessels, circulating blood, and lymphoid organs, systemic inflammation contributes to initiating but also to aggravating the stroke response [9-11].

Despite the increase in white blood cells (WBCs) count in AIS patients has been largely associated with mortality [12], the clinical relevance of systemic inflammatory biomarkers in HT has been addressed without considering the influence of infections, mostly in single-center studies with limited sample sizes and patients’ heterogeneity; thus, its predictive value remains largely unknown. Therefore, in this study we sought to explore the relationship of these variables using a large-scale database, the National Inpatient Sample (NIS).

## Materials and methods

Data source

This retrospective cohort study used the NIS database. This large database contains a 20% stratified sample of all hospital discharges in states participating in the Healthcare Cost and Utilization Project (HCUP) in the U.S. It contains demographic, diagnostic, and procedural data, with diagnoses and procedures captured in the form of the International Classification of Diseases 10th edition (ICD-10).

Study sample

Primary inclusion criteria were patients with Stroke (ICD-10th: I66) from January 1, 2016 to December 31, 2017. Exclusion criteria comprised: 1) patients under 18 years old and/or 2) patients with a diagnosis of gastrointestinal, urogenital, respiratory infection, bacteremia, viral infection, sepsis, or fever.

Collected data

Examined data comprised demographics including age, sex, race, and median household income categorized in quartiles. Comorbidities analyzed included hypertension, diabetes mellitus with and without complications, congestive heart failure, renal disease, myocardial infarction, chronic pulmonary disease, peripheral vascular disease, status post-administration of recombinant tissue plasminogen activator (rtPA), thrombectomy, National Institute of Health Stroke Scale (NIHSS) Score and history of cerebrovascular disease (see Appendix).

Finally, we selected the most prevalent pro-inflammatory WBC anomalies found in the NIS 2016 and 2017 database, this included eosinophilia, lymphocytosis, monocytosis, plasmacytosis, bandemia, and elevated WBC count (leukocytosis). All codes for this study are shown in the supplementary material (see Appendix).

Outcomes

The primary outcome analyzed for patients with AIS was the development of hemorrhage transformation (HT) (ICD-10th: I61-I62.9). Our secondary outcome analyzed was death during hospitalization (in-hospital mortality).

Statistical analysis

Interactions between specific types of pro-inflammatory WBC anomalies and outcomes were assessed. A univariate analysis was done to identify unevenly distributed variables in the WBC anomalies that could be playing as confounding factors; for this step, all continuous values were represented using mean ± standard deviation (SD) or median and interquartile range (IQR). Categorical variables were described using frequencies and proportions. Comparisons were performed using Student’s t-test, the nonparametric Mann-Whitney test or χ^2^ tests as appropriate.

Factors with a p-value less than 0.10 were then included in a multiple logistic regression analysis to reflect the adjusted association between WBC anomalies and each of the primary outcomes. Odds ratios (OR) and 95% confidence intervals (CI) were calculated. A p-value less than 0.05 was considered statistically significant. SPSS v.24 software (IBM, Armonk, NY) was used to perform the statistical analysis.

## Results

**Figure 1:**
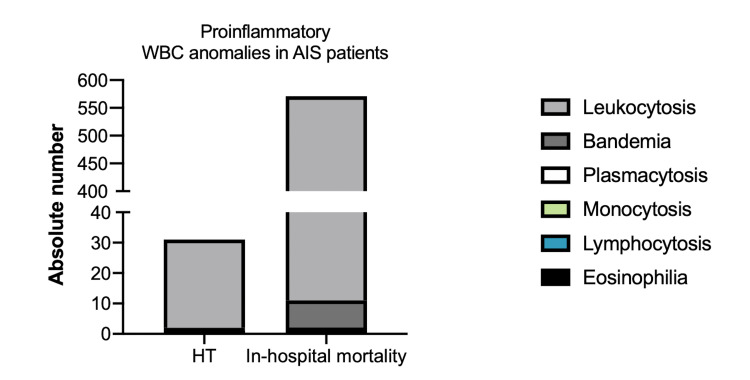
Proinflammatory white blood cell (WBC) anomalies in AIS patients. Total number of AIS patients found in the NIS 2016 and 2017 with WBC anomalies and with hemorrhagic transformation (HT) and/or died during hospitalization. AIS - Arterial ischemic stroke

A total of 260,189 patients with AIS were captured in the 2016 & 2017 NIS database. Out of these patients, 47,833 (18.4%) were excluded due to a diagnosis related to fever or infections. Therefore, 212,356 patients were included for the main analysis. We identified 422 (0.2%) patients with HT, and 10,230 (4.8%) that died during hospitalization. At least one of the proinflammatory WBC parameters were found in 7.3% (n=31) of the HT patients and in 5.6% (n=571) of patients that died during hospitalization, the most prevalent anomaly was leukocytosis with 6.9% (n=29) and 5.5% (n=560) in HT and died during hospitalization, respectively (Figure 1).

0.2% of patients had eosinophilia and 0.2% had bandemia in the HT cohort, while in patients that died in-hospital 0.02% had eosinophilia and 0.09% bandemia. The rest of WBC anomalies represented the 0% of the cases.

 

We proceed with a univariate analysis to assess unevenly distributed variables in patients with normal WBC and leukocytosis (Table 1).

**Table 1:** Univariate analysis of patients with/without leukocytosis in the NIS database, 2016 and 2017. *Included in multivariate analysis NIS - National inpatient sample

Variables	White Blood Cells Count		P-value
	Leukocytosis (n= 7,307)	Normal (n= 205,046)	
Age in years at admission, mean (SD)	67 (15)	69 (15)	<0.001*
Female, n (%)	3,488 (47.7)	97,674 (47.6)	0.868
Race, n (%)			
White	5,049 (71.8)	135,801 (68.7)	<0.001*
African-American	927 (13.2)	34,199 (17.3)	
Hispanic	603 (8.6)	15,797 (8)	
Others	457 (6.5)	11,734 (6)	
Household income, n (%)			
Quartile 1	2,206 (30.7)	62,494 (31)	0.787
Quartile 2	1,904 (26.5)	52,531 (26.1)	
Quartile 3	1,723 (24)	48,036 (23.6)	
Quartile 4	1,354 (18.8)	38,543 (19.1)	
Hypertension, n (%)	5,451 (74.6)	152,305 (74.3)	0.537
Diabetes simple, n (%)	2,231 (30.5)	61,994 (30.2)	0.586
Diabetes complicated, n (%)	779 (10.7)	24,981 (12.2)	<0.001*
Congestive heart failure, n (%)	1,373 (18.8)	38,720 (18.9)	0.841
Renal disease, n (%)	1,282 (17.5)	38,720 (18.1)	0.262
Myocardial infarction, n (%)	949 (13.0)	22,282 (10.9)	<0.001*
Chronic pulmonary disease, n (%)	1,286 (17.6)	33,949 (16.6)	0.019*
Peripheral vascular disease, n (%)	846 (11.6)	21,556 (10.5)	0.004*
Status post administration of tPA, n (%)	283 (3.9)	5,903 (2.9)	<0.001*
Thrombectomy, n (%)	458 (6.3)	8,478 (4.1)	<0.001*
NHISS score, n (%)			
0-9	664 (9.1)	2,3710 (11.6)	<0.001*
10-19	284 (3.9)	5,064 (2.5)	<0.001*
20-29	159 (2.2)	2,203 (1.1)	<0.001*
30-39	31 (0.4)	306 (0.1)	<0.001*
40-42	1 (0.0)	19 (0.0)	0.702
Localization, n (%)			
Extracranial			
Unspecified	59 (0.8)	1,611 (0.8)	0.836
Vertebral artery	140 (1.9)	2,878 (1.4)	<0.001*
Basilar artery	89 (1.2)	2,125 (1.0)	0.133
Carotid artery	467 (6.4)	10,918 (5.3)	<0.001*
Intracranial			
Unspecified	409 (5.6)	11,531 (5.6)	0.924
Middle cerebral artery	1,856 (25.4)	38,824 (18.9)	<0.001*
Anterior cerebral artery	155 (2.1)	3,001 (1.5)	<0.001*
Posterior cerebral artery	1,856 (25.4)	38,824 (18.9)	<0.001*
Cerebellar	259 (3.5)	5,286 (2.6)	<0.001*

Variables with p < 0.1 included age, race, complicated diabetes mellitus, myocardial infarction, chronic pulmonary disease, peripheral vascular disease, thrombolysis with rtPA, patients with NIHSS score, and vertebral artery, carotid artery, middle cerebral artery, anterior cerebral artery, posterior cervical artery, and cerebellar localizations. All mentioned variables were included to adjust the multiple logistic regression models.

**Figure 2:**
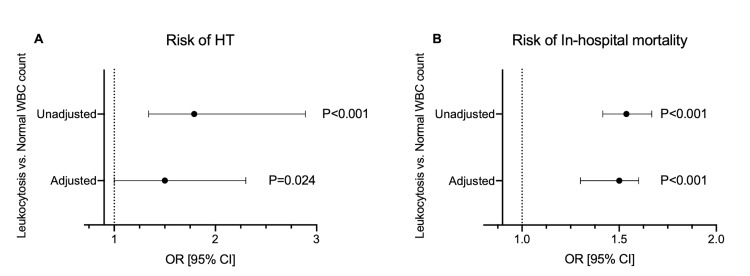
Risk of hemorrhagic transformation (HT) and in-hospital mortality in patients with leukocytosis. (A) Crude and adjusted odds ratio (OR) with 95% confidence interval (95% CI) of leukocytosis with risk of HT following AIS. (B) Crude and adjusted OR with 95% CI of leukocytosis with risk of in-hospital mortality in AIS patients. AIS - Arterial ischemic stroke

After adjusting for variables with p < 0.1 in the univariate analysis, the multiple logistic regression model revealed that leukocytosis is an independent risk factor for developing HT [OR = 1.5, 95% CI: 1-2.3, p=0.024] as well as increased the risk of in-hospital mortality [OR = 1.5, 95% CI: 1.3-1.6, p <0.001] in AIS patients (Figures 2A, 2B).

.

## Discussion

In our study, we analyzed the association of systemic pro-inflammatory WBC markers and HT in patients with AIS. We found that leukocytosis increases 1.5-fold times the risk of HT independently of socio-demographic, comorbidities, and treatment factors. Furthermore, our results support previous evidence regarding the higher risk of in-hospital mortality in AIS with leukocytosis. In addition, our analyses excluded patients coursing urinary tract infections, lung infections, gastrointestinal infections, sepsis, and/or fever. Therefore, we report an adjusted large national-scale study with important implications for physicians taking care of AIS patients that are not coursing an active infection or secondary cause of systemic inflammation.

Despite HT is a serious complication commonly following reperfusion therapy and, sometimes as a result of the natural evolution of the disease [13], the current treatment recommendations include anticoagulants, thrombolysis, and intravascular procedures [14]. Thus, available and routine clinical prediction tools of HT based on pathophysiological mechanisms are necessary to decide the best management options according to the patient’s system response to stroke. In this context, experimental studies have clarified that WBC has a critical role in post-ischemic neuroinflammation and secondary neurovascular injury [15]. Within 4 to 6 h after AIS onset, circulating leukocytes adhere to the vessel wall, transmigrate through the BBB and enter the brain, subsequently releasing pro-inflammatory mediators, resulting in a secondary lesion in the penumbra surrounding the nucleus of the ischemia [8]. Neutrophils are the earliest WBC subtype to infiltrate the ischemic brain [16]. These immune cells exacerbate the injury by releasing additional inflammatory factors, such as ROS, MMP-9, and neutrophil elastase, which are prominent effectors of BBB disruption [17]. It also has been shown that most AIS patients course some degree of immune dysregulation; however, when these mechanisms are overstimulated worsen short and long-term clinical outcomes are expected [18-20].

Regarding the clinical evidence in this matter, in spite most of the reports that have shown an association of leukocytes subtypes with increased risk of HT have not taken into account infections, a recent study by Semerano et al. [21] showed that increasing leukocytes predicts worsen outcomes post-AIS, including HT independently of preceding or early post-stroke infections, suggesting that stroke-evoked sterile inflammation is a major key to understand patients’ hospital course and prognosis. Even though in the mentioned study the authors treated the WBC as continuous variables, their findings showed that high leukocyte counts increase 1.4 times the odds of HT, which is in line with our findings. In addition, our results showed an increased risk of in-hospital mortality in patients with leukocytosis which supports previous studies [12,22] and hence, strongly suggests that AIS patients with evidence of sterile inflammation may benefit from complementary management therapies such as closer monitoring and anti-inflammatories from early stages.

Although we consider our results are encouraging since it is the first demonstration, to our best knowledge, of the potential suggestive value of a routine laboratory marker for a serious complication in AIS using a large-national database, limitations inherent to the retrospective nature of our work need to be mentioned. Our variables of interest, WBC, are presented in the NIS database as categorical variables instead of continuous values and therefore, it was not possible to identify a threshold that could increase the sensitivity and specificity to predict HT and mortality. Moreover, this study does not elucidate the essential causes and mechanisms by which systemic inflammation put in a higher risk of worsening outcomes. Thus, further clarification of mechanisms, cut-off values, and specific leukocyte subtypes is needed.

## Conclusions

Peripheral leukocytosis is an independent predictor of HT and in-hospital mortality in AIS patients that did not course with a pre/post-AIS infection or other sources of systemic inflammation besides the attributed AIS-induced inflammation. Hence, this national study supports previous experimental and clinical evidence regarding the role of sterile inflammation in stroke pathophysiology and as a potential predictive biomarker. Nevertheless, further validation regarding the dynamic change of WBC during the acute and subacute post-AIS phases and the role of other proinflammatory molecules are needed to incorporate the concept of sterile leukocytosis in clinical reasoning.

## Appendices

**Table 2:** ICD-10 codes used for the analysis of peripheral white blood cell abnormalities in AIS patients discharged in the NIS 2016 and 2017 dataset. ICD-10 - International Classification of Diseases AIS - arterial ischemic stroke NIS - National Inpatient Sample

Variables included	ICD-10
Arterial ischemic stroke	I66
Hemorrhagic transformation	I61-I62.9
Eosinophilia	D72.1
Lymphocytosis (symptomatic)	D72.820
Monocytosis (symptomatic)	D72.821
Plasmacytosis	D72.822
Bandemia	D72.825
Elevated white blood cell count	D72.828, D72.829
Hypertension	I10
Diabetes mellitus simple	E10, E11, E12, E13, E14
Diabetes mellitus complicated	E10, E11, E12, E13, E14
Lipidemias	E78
Obesity	E66, Z683, Z684,
Congestive heart failure	I099, I110, I13, I255, I42, I43, I50, P290
Cerebrovascular disease	G45, G46, H340, I6
Depression	F32, F33
Current smoker	F17
Sleep Apnea	G47.3
Status post-administration of recombinant tissue plasminogen activator (rtPA)	Z92.82
Thrombectomy	037J3, 037G3
National Institute of Health Stroke Scale (NIHSS)	R29.7
